# Plants from Arid and Semi-Arid Zones of Mexico Used to Treat Respiratory Diseases: A Review

**DOI:** 10.3390/plants13060792

**Published:** 2024-03-11

**Authors:** Irma E. Dávila-Rangel, Ana V. Charles-Rodríguez, Julio C. López-Romero, María L. Flores-López

**Affiliations:** 1 Universidad Interserrana del Estado de Puebla Ahuactlán, Ahuacatlán 73330, Mexico; idavilarangel@gmail.com; 2 Departamento de Ciencia y Tecnología de Alimentos, Universidad Autónoma Agraria Antonio Narro, Saltillo 25315, Mexico; 3 Departamento de Ciencias Químico-Biológicas y Agropecuarias, Universidad de Sonora, Caborca 83600, Mexico; julio.lopez@unison.mx; 4 Centro de Investigación e Innovación Científica y Tecnológica, Universidad Autónoma de Coahuila, Saltillo 25070, Mexico

**Keywords:** medicinal plants, bioactive compounds, respiratory diseases, ethnopharmacology, pharmacological properties

## Abstract

Medicinal plants have been a traditional remedy for numerous ailments for centuries. However, their usage is limited due to a lack of evidence-based studies elucidating their mechanisms of action. In some countries, they are still considered the first treatment due to their low cost, accessibility, and minor adverse effects. Mexico is in second place, after China, in inventoried plants for medicinal use. It has around 4000 species of medicinal plants; however, pharmacological studies have only been carried out in 5% of its entirety. The species of the Mexican arid zones, particularly in semi-desert areas, exhibit outstanding characteristics, as their adverse growing conditions (e.g., low rainfall and high temperatures) prompt these plants to produce interesting metabolites with diverse biological activities. This review explores medicinal plants belonging to the arid and semi-arid zones of Mexico, focusing on those that have stood out for their bioactive potential, such as *Jatropha dioica*, *Turnera diffusa*, *Larrea tridentata*, *Opuntia ficus-indica*, *Flourensia cernua*, *Fouquieria splendes*, and *Prosopis glandulosa*. Their extraction conditions, bioactive compounds, mechanisms of action, and biological efficacy are presented, with emphasis on their role in the treatment of respiratory diseases. Additionally, current research, novel applications, and perspectives concerning medicinal plants from these zones are also discussed.

## 1. Introduction

Throughout the history of humanity, traditional medicine has been the remedy for the cure of many diseases. Thanks to previous generations, humanity has knowledge about the use and effect of various medicinal plants. The most ancient knowledge of herbal medicine is found in Traditional China Medicine, which has more than 5000 years of knowledge of 365 drugs [1]. Over time, different cultures have documented their knowledge of medicinal plants. In Mexico, there is the Codex de la Cruz-Badiano, written by a Nahuatl physician and translated into Latin by Juan Badiano, which describes 227 medicinal plants, most of them of autochthonous origin [2]. 

**Figure 1:**
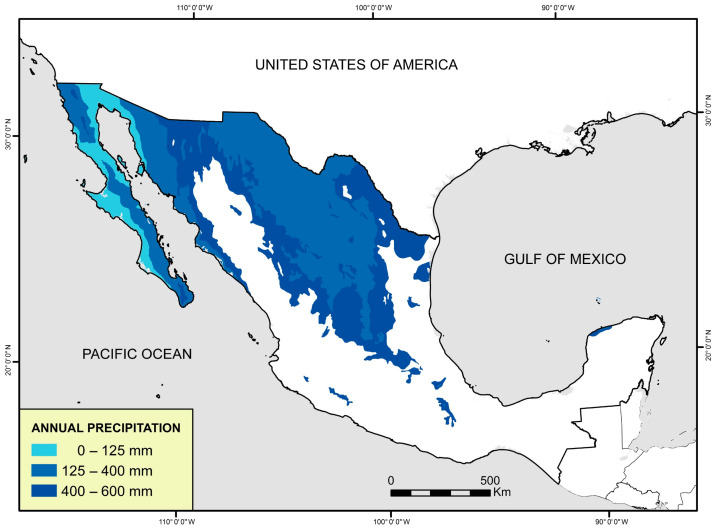
Arid and semi-arid zones of Mexico. Map of Mexico in blue color shows the very dry, dry, and semi-dry climates of Mexico according to the average annual precipitation.

Mexico ranks fifth in the plant diversity and second place in the world in registration of medicinal plants. It has between 21,073 and 23,424 vascular plants that have been classified to date. On the other hand, around 4000 plant species with medicinal attributes are estimated to exist in Mexico, representing 15% of the total Mexican flora [3]. The arid lands cover 60% of its surface. In a semi-arid zone, annual rainfall is between 250 and 500 mm (Figure 1) [4]. The regions with arid and semi-arid zones in Mexico are Chihuahua, Coahuila, Durango, Zacatecas, San Luis Potosí, Nuevo León, Tamaulipas, Baja California, and Sonora [5,6]. These areas have a wide range of flora adapted to hostile environments of low rainfall and high temperatures [7]. Among the arid and semi-arid zones plant species in Mexico, certain ones stand out for their bioactive potential and characteristics: *Jatropha dioica*, *Flourensia cernua*, *Turnera diffusa*, *Eucalyptus camaldulensis*, *Euphorbia antisyphilitica*, *Larrea tridentata*, *Lippia graveolens*, *Opuntia ficus-indica*, *Agave* spp., *Prosopis glandulosa*, *Punica granatum*, *Eysenhardtia texana*, *Machaeranthera pinnatifida*, *Mentha piperita*, *Rhus microphylla*, *Notholaena sinuata*, *Gnaphalium* spp., *Sambucus nigra*, *Matricaria chamomilla*, and *Populus fremontii* [8,9,10,11,12]. 

Medicinal plants are rich in secondary metabolites, and depending on the genera and species, they may contain different chemical classes and concentrations in different parts of the plant. Many plant species in the arid and semi-arid zones of Mexico have been studied for their bioactive compounds, which are produced in response to the climatic conditions that promote the synthesis of secondary metabolites [13,14]. When plants experience stress, such as exposure to certain environmental factors like heat, cold, drought, salinity, or specific pathogens, they often activate various defense mechanisms [14,15,16]. The bioactive compounds present in medicinal plants can vary widely in nature, including polyphenols, flavonoids, terpenoids, alkaloids, glycosides, waxes, vitamins, and fatty acid esters. These compounds may serve different biological functions that benefit the plants, such as defense against pathogen or insect attack, pollinator attraction, resistance to adverse weather conditions, among others [7,17,18]. 

On the other hand, some of these metabolites have important effects at the cellular and physiological level in the human organism, owing to their therapeutic properties. They have effects on different diseases, including respiratory diseases, neurodegenerative diseases, cardiovascular diseases, diabetes, obesity, among others [18,19,20,21,22,23].

Respiratory tract infections are one of the most common in the world. They mainly include chronic obstructive pulmonary disease (COPD), asthma, pneumonia, pulmonary fibrosis, lung cancer, and, more recently, SARS-CoV-2 (COVID-19). Most of the population suffers from some respiratory disease at least once a year [19,24]. In 2019, the entire world was affected by the SARS-CoV-2, a virus that affects the respiratory system and caused the death of millions of people during the pandemic. One of the alternatives to improve the symptoms caused by COVID-19 is the use of medicinal plants. However, there is limited information on the study of arid and semi-arid zones of Mexico to treat respiratory diseases, which needs further investigation for designing novel herbal formulations [19]. To date, only *Opuntia ficus-indica* belonging to the semi-arid zones of Mexico has been studied for its interaction between chiral compound astragalin with SARS-CoV-2 Mpro, like a possible protease inhibitor, that is one of the most investigated protein targets of therapeutic strategies for COVID-19 [25]. 

The plants *Jatropha dioica*, *Turnera diffusa*, *Larrea tridentata*, *Opuntia ficus-indica*, *Flourensia cernua*, *Fouquieria splendes*, and *Prosopis glandulosa* have long been utilized in traditional medicine within the arid and semi-arid zones of Mexico, including the Chihuahua Desert and the Sonoran Desert. They are employed to alleviate a variety of respiratory ailments such as bronchitis, asthma, colds, and coughs. Therefore, this review discusses the main bioactive compounds of some plants from arid and semi-arid zones of Mexico, their extraction techniques, their effectiveness against certain respiratory diseases due to their scientifically proven pharmacological properties, which can be targeted by therapies for their anti-inflammatory, antimicrobial, and anticancer characteristics.

## 2. Respiratory Diseases

Respiratory diseases can be caused by microbial infections, environmental pollutants, allergens, and genetic predisposition mainly affecting the lungs, some of the symptoms can cause chest discomfort, wheezing, flu, cough, weakness, and other symptoms. Respiratory diseases can be acute or chronic, untreated can cause further discomfort or be fatal. Infections caused by fungi, viruses or bacteria can affect the respiratory tract and the defense system is activated by sending defense cells such as lymphocytes, monocytes, and neutrophils to the site of infection. Nevertheless, the hyperproduction of neutrophils causes the production of Reactive Oxygen Species (ROS), resulting in oxidative stress and inflammation that can compromise the lung’s defense system [26]. Currently, conventional therapy offers a wide range of treatments for respiratory diseases. However, certain drugs designed to interact with multiple targets have been linked to adverse side effects such as diarrhea, liver dysfunction, nausea, loss of appetite, and vomiting [27]. Conversely, studies have shown that combining conventional treatment with the use of medicinal plants can improve symptoms in patients with respiratory diseases caused by SARS-CoV-2, without adverse reactions [28]. One of the significant challenges identified by the World Health Organization (WHO) is the urgent need to explore alternatives to combat antimicrobial resistance associated with conventional therapies. Without intervention, the WHO estimates that drug-resistant diseases could result in 10 million deaths annually by 2050. Therefore, it is crucial to investigate alternatives such as medicinal plants therapies, either alone or in combination with conventional drugs, to address the impending threat [29]. Antioxidant therapy or antioxidant-rich diet is a good option to counteract lung diseases, including those associated with SARS-CoV-2, to reduce oxidative stress in the respiratory system [20,30]. In this sense, medicinal plants are a form of natural therapy that can counteract or prevent various diseases of the respiratory tract, due to their bioactive compounds, such as polyphenols. Table 1 shows the factors that contribute to respiratory diseases. In addition to the factors mentioned in this table, emotional conditions such as anxiety, stress, and depression can exacerbate respiratory diseases.

In several countries, herbal remedies offer a cost-effective therapy to treat illnesses at a low cost. While Mexico generally has access to medical services, certain rural areas still prefer natural remedies for diverse ailments, a practice observed throughout the country. In Mexico’s semi-desert regions, specific plants, such as *J. dioica*, *T. diffusa*, *L. tridentata, F. cernua*, among others, are recognized for their bioactive compounds with antiviral, antibacterial, and antioxidant properties, potentially inhibiting the growth of pathogenic microorganism responsible for pneumonia, tuberculosis, and viral flu [46,47,48,49].

## 3. Scientific Evidence of Semi-Desert Plants Used to Treat Respiratory Diseases

Globally, one of the main causes of concern in hospitals is respiratory tract infections caused by opportunistic bacteria that acquire resistance to antibiotics. Chronic obstructive pulmonary disease and asthma were the two leading causes of death in Mexico, with a total of 21,441 deaths recorded in people over 75 years of age [50]. However, after the pandemic caused by the SARS-CoV-2 virus, which causes respiratory diseases, the number of deaths increased to 334,107 in Mexico [51]. 

Bioactive compounds are chemical compounds that vary according to each plant genus and species, and various biotic or abiotic factors are involved in their expression and concentration. Among the isolated bioactive compounds of medicinal plants with recognized antiviral action are rutin (known as quercetin-3-rutinoside), a flavonoid glycoside effective against avian influenza virus and parainfluenza -3 virus. Quercetin, an aglycone of rutin, has demonstrated its therapeutic potential against influenza A virus (IFV-A), rhinovirus, dengue virus type 2, poliovirus type 1, adenovirus, Epstein–Barr virus, Mayaro virus, Japanese encephalitis virus, and RSV [21].

**Figure 2:**
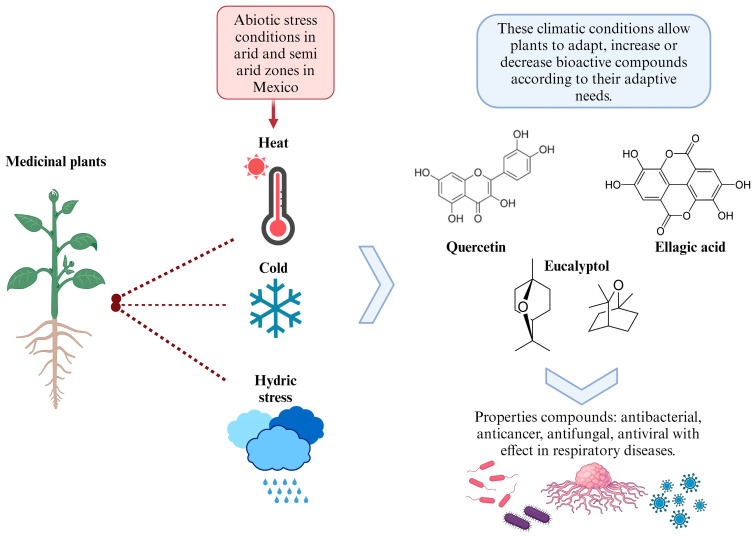
Effect of plants from arid and semi-arid zones of Mexico against abiotic stress.

The following section highlights medicinal plants from the arid and semi-arid zones of Mexico known for their role in the treatment of respiratory diseases. Figure 2 provides an overview of these medicinal plants and illustrates how the abiotic stresses, such as extreme temperatures and water scarcity, can influence the synthesis of secondary metabolites. These stressors may either enhance or diminish the production of metabolites, often favoring and increasing compounds with antioxidant properties. This increase in antioxidants can directly impact respiratory diseases, which will be explored in the subsequent sections.

### 3.1. Jatropha dioica

It is commonly known as “Sangre de Drago”. The habitat of this species is in regions with dry and semi-dry climates. It is in Texas, USA, and the North of Mexico, although it can be found in all the Mexican territory [52]. The genus *Jatropha* (Euphorbiaceae) has about 200 species of woody trees, shrubs, subshrubs, or herbs. It is a shrub 50 cm to 1.50 m high. It owes its common name to the fact that it has a colorless juice that changes to dark when in contact with air. Its branches are reddish-brown with leaves longer than wide. Its flowers are small and in clusters of pink. The globose fruits are 1.5 cm long and have a seed [53]. *Jatropha* species are used in traditional medicine to treat many clinical conditions, such as stomach pain, toothache, bloating, inflammation, leprosy, dysentery, dyscrasia, vertigo, anemia, diabetes, as well as to improve human immunodeficiency virus conditions, tumors, ophthalmia, ringworm, ulcers, malaria, skin diseases, bronchitis, asthma, and as an aphrodisiac [54]. Several studies have demonstrated the antimicrobial effectiveness of *J. dioica*, although it depends on the solvent and the part of the plant used. The methanol extract of *J. dioica* was effective against *Staphylococcus aureus* and *Klebsiella pneumonia*, which presented bioactive compounds such as flavonoids, terpenes/sterols, lactons, and quinons [49]. Another study showed that organic extracts of *J. dioica* root, prepared with hexane, ethanol, and acetone, evaluated on human pathogens such as *Bacillus cereus*, *Escherichia coli*, *Salmonella typhi*, *S. aureus*, *Enterobacter aerogenes*, *E. cloacae*, *S. typhimurium*, *Cryptococcus neoformans*, *Candida albicans*, *C. parapsilosis*, and *Sorothrix schenckii*, had a high antibacterial and antifungal activity that may come from the presence of β-sitosterol (terpene) compounds, which was extracted using hexane [55]. Some of these microorganisms are pathogens that cause respiratory diseases, such as *S. aureus*, *Pseudomona aeruginosa*, *Haemophylus influenzae*, *Streptococcus pneumoniae*, *E. coli* [56], among many other strains. In general, different extracts of this plant have shown antimicrobial properties, tested against strains involved in respiratory diseases, proving its specific effectiveness by the bioactive compounds found.

### 3.2. Turnera diffusa

*T. diffusa* was one of the most important medicinal plants at the time of the Maya culture. Commonly known as “damiana”, a shrub that grows in the arid and semiarid regions of South America, Mexico, USA, and the West Indies. *T. diffusa* is a shrub up to 1.5 m high that is very branched and whose stems are slightly reddish. The leaves are small, wrinkled and have teeth on the edge; they give off a strong aroma when squeezed. Its flowers are yellow and look like little stars hidden among the branches and its fruits are capsules. It is one of the most highly appreciated plant aphrodisiacs. In addition, it was also used against cerebral weakness, impotence, and orchitis [47]. It is a medicinal plant studied for its pharmacological action and their properties antioxidant and antimicrobial. The main bioactive compounds of *T. diffusa* include flavonoids, phenolic acids and derivatives, cyanogenic derivates, fatty acids, alkaloids, terpenoids, and sugars conjugates. In a study reported by Urbizu et al. [57], 21 compounds were identified in essential oils of *T. diffusa* including Ethanone, 1-(1,3-dimethyl-3-cyclohexen-1-yl) and Eucalyptol. This last is known to have antibacterial properties, thus promoting the elimination of bacteria from tobacco-exposed lungs by attenuating ciliated cell damage and suppressing the expression of the MUC5AC gene, a protein that is related to mucus hypersecretion in the pulmonary tract [58]. Thirty-five bioactive compounds classified within the above compounds were detected in *T. diffusa*, including five new compounds detected in this plant and one new compound (6) not previously studied: luteolin 8-C-E-propenoic acid (1), luteolin 8-C-β-[6-deoxy-2-*O*-(α-L-rhamnopyranosyl)-xylo-hexopyranos-3-uloside] (2), apigenin 7-O-(6″-*O*-*p*-Z-coumaroyl-β-D-glucopyranoside) (3), apigenin7-*O*-(4″*-O*-*p*-Z-coumaroyl-glucoside) (4), syringetin 3-*O*-[β-D-glucopyranosyl-(1→6)-β-D-glucopyranoside] (5), and laricitin 3-*O*-[β-D-glucopyranosyl-(1→6)-β-D-glucopyranoside] (6) [48]. A study showed that *T. diffusa* has bactericidal, inhibitory, and bacteriostatic action at Minimal Inhibitory Concentration (MIC) of 300 µg/mL doses against *S. aureus* [59]. This bacterium has multiple virulence factors that give it the ability to penetrate the body’s barriers and produce diseases such as pneumonia [60]. On the other hand, it is known that the medicinal bioactivity and the industrial interest may be affected depending on the locality of collection of this plant, because despite having many bioactive compounds, the presence, or concentrations of these may vary. Despite this, *T. diffusa* has multiple beneficial properties for human health [57].

### 3.3. Larrea tridentata

Commonly known as “gobernadora” or “creosote bush”, is a bush from the northern region of Mexico and the south of the USA. It is a very branched shrub, evergreen, from 0.6 to 3 m high. Leaves formed by two leaflets joined together at the base to each other at the base. Fruit subglobose to obovoid, 7 mm long, with a long. This vegetal specimen possesses a wide variety of secondary metabolites such as lignans, flavonoids, glycosylated flavonoids, saponins, sterols, tannins, terpenes, and essential oils [61]. Among the most promising and widely reported metabolites from *L. tridentata*, are as follows: 3′-demethoxy-6-*O*-demethylisoguaiacin, 3′-*O*-methyldihydroguaiaretic acid, meso-dihydroguaiaretic acid, and tetra-*O*-methylnordihydroguaiaretic acid. These have been reported to exhibit antibacterial, antiprotozoal, anthelmintic, antifungal, antiviral, anticancer, and antioxidant activities [62]. Various studies demonstrate the biological effectiveness of this plant in diseases such as: antidiabetic, anti-inflammatory, antibacterial, antituberculosis, antifungal, antiviral activities. Núñez-Mojica [61] showed that chloroform extract of *L. tridentata*, isolated and characterized two new cyclolignans; one of them proved to have an effect against *Mycobacterium tuberculosis*. Another study by Favela-Hernández [63] involved the extraction of dried and ground leaves using chloroform, and tested the effectiveness of several bioactive compounds against pathogenic bacteria of the respiratory tract: dihydroguaiaretic acid had activity towards methicillin resistant *S. aureus* (MRSA) (MIC 50 µg/mL) and multidrug-resistant (MDR) strains of *M. tuberculosis* (MIC 12.5–50 µg/mL); 4-epi-larreatricin was active against MDR strains of *M. tuberculosis* (MIC 25 µg /mL); 3′-Demethoxy-6-*O*-demethylisoguaiacin displayed activity against sensitive and resistant *S. aureus* (MIC 25 µg/mL) and MDR strains of *M. tuberculosis* (MIC 12.5 µg /mL). 5,4′-Dihydroxy-3,7,8,3′-tetramethoxyflavone and 5,4′-dihydroxy-3,7,8-trimethoxyflavone were active against *M. tuberculosis* MDR strains having MIC values of 25 and 25–50 µg/mL, respectively, while 5,4′-dihydroxy-7-methoxyflavone was active against *S. aureus* (MIC 50 µg/mL). *L. tridentata* has a great antibacterial potential against respiratory tract diseases, specifically against tuberculosis, but further studies are needed to evaluate possible adverse effects.

### 3.4. Opuntia *spp.*

In Mexico, the genus *Opuntia* has a wide distribution. Wild species of the genus *Opuntia* are part of the natural ecosystems of semi-arid regions [64]. The regions with the greatest species richness are the central and northern Altiplano, the northwest, the Bajío, and the Tehuacán-Cuicatlán Valley. The subfamily Opuntioideae is characterized by the presence of fugacious, cylindrical, ribless leaves; glochids on the areoles; a hard, generally light-brown aril covering the campylotropic ovule. The arid and desert regions of Mexico are inhabited by the largest number of *Opuntia* species in the world. There are endemic species of great importance like: *O. engelmannii*, *O. macrocentra*, *O. durangensis*, *O. phaeacantha*, *O. rastrera*, and *O. microdasys* [3]. 

The endemic species of the north of the country are those that resist frost, usually have thin stalks, which prevents them from dying when the water they contain freezes. Extracts from *Opuntia* species contain phenolic compounds, other antioxidants such as ascorbate, pigments such as carotenoids and betalains, and other phytochemicals [65]. Elkady et al. (2020) [66] characterized a fraction extracted in ethyl acetate from the prickly pear fruit peel, the authors detected a quercetin 5,4′-dimethyl ether, which exhibited an inhibitory effect against pneumonia pathogens. *Opuntia* flower extracts also showed in vitro antibacterial activity against *P. aeruginosa*, *S. aureus*, *B. subtilis*, and antifungal activity against *C. lipolytica*, and *Aspergillus niger* [65]. Regarding respiratory diseases caused by viruses or bacteria, it is precisely the plants of the *Opuntia* genus, which have been proven effective against several pathogenic strains that attack the respiratory tract.

### 3.5. Flourensia cernua

Commonly known as hojasen, it is a shrub that grows in the desert and semi-arid areas of the southern USA and northern Mexico such as Chihuahua, Sonora, Coahuila, Durango, Nuevo León, San Luis Potosi, and Zacatecas. It is a plant known for its use in traditional medicine for the treatment of gastrointestinal diseases such as stomach pain, diarrhea, expectorant, and anti-rheumatic [67,68]. It grows to a height of 1 to 2 m. Branches covered with thick, oval leaves up to 2.5 cm long. The hanging flower heads contain several yellow disk florets. The fruit is a hairy achene up to 1 cm long. Most parts of the plant are highly resinous and have a tarry or hoppy odor with a bitter taste [67]. The people consume this plant in infusion and decoction of leaves and steams [10]. Its bioactive molecules find extensive applications in the agri-food and health industries. 

One of most frequent types of cancer in Mexico is lung cancer, more than 7500 new cases and 7100 deaths associated with this neoplasia were registered, being the seventh cancer with the highest incidence and the fourth cause of death from cancer in this country [69]. A study showed that three extracts of *F. cernua*, *F. retinophylla*, and *F. microphylla*, have a high content of total phenolic compounds, and presented antiproliferative effect on A549 cells, which is a lung epithelial cell line derived from human alveolar cell carcinoma. Besides, *F. cernua* demonstrated the best anti-inflammatory activity, therefore these extracts can be used for the control of lung cancer [18]. A study employing a hexane extract of *F. cernua* reported a MIC of 50 and 25 μg/mL against sensitive and resistant strains, effectively inhibiting and eradicating *M. tuberculosis* growth [70]. Therefore, *F. cernua* can be a target for therapies against lung cancer; case studies are required to verify its effectiveness in different stages of this type of cancer.

### 3.6. Fouquieria splendes

Commonly known as “ocotillo”, is a medicinal plant endemic to the arid and semi-arid zones of the southwestern United States and northern Mexico, distributed throughout the deserts of Sonora and Chihuahua, and is one of the most representative species of the Fouquieriaceae family (thirteen species) [71,72]. The plant grows as a shrub with thorny stems reaching 2 to 10 m and it is characterized by having leaves after rain. In addition, this plant presents inflorescences made up of flowers that can vary from white to red-orange, usually appearing during spring [73,74]. This plant is used in folk medicine as bark tincture from bark, infusion of the stem, roots, leaves, and flowers, or the direct use of leaves and flowers. These are used against different health conditions, such as cough, congestion, infections, stomach pain, wounds, painful, pelvic circulation, hemorrhoids, prostatic hyperplasia, and menstrual cramps [75,76]. On the other hand, scientific studies have shown that extracts from this plant exhibited antiproliferative, antioxidant, insecticidal, antiparasitic, antifungal, and antimicrobial potential [14,71,73,75,77]. Regarding the antimicrobial effect, some research has been developed. However, this plant has been shown to have an effect against microorganisms associated with respiratory tract infections, such as *S. aureus*. A study performed by Vega Menchaca et al. [75] demonstrated that a methanol extract of *F. splendens* leaves affected the growth of *S. aureus*, showing a MIC value of 25 µg/mL. Similarly, Rodríguez Garza [73] validated that a methanol stem extract inhibited *S. aureus* growth, showing inhibition halos greater than 20 mm at a concentration of 100 mg/mL. In addition to the above, various investigations have been focused on the antimicrobial analysis of a compound isolated from *F. splendens* called ocotillo, which is a triterpene, chemically modified with antimicrobial activity against bacteria associated with respiratory infections such as *S. aureus, P. aeruginosa*, and *K. pneumoniae* ATCC and resistant clinical isolates [78,79]. On the other hand, it is important to mention that the extract of *F. splendens* also presents antifungal and antiviral effects against microorganisms not associated with respiratory tract infections [73,80]. The above opens the possibility of analyzing the effect of *F. splendens* against microorganisms of clinical interest. On the other hand, in addition to triterpenes, other chemical compounds associated with the antimicrobial potential of *F. splendens* are phenolic compounds, identifying various flavonoids, phenolic acids, and anthocyanins, as well as fatty acids, which have presented antimicrobial effect through in vitro and in vivo tests [14,71,72,74,81]. An important aspect to highlight is that the *F. splendens* extract demonstrated low cytotoxicity in the in vitro evaluation with cell lines, in addition to showing low in vivo cytotoxicity against *Artemia salina* when exposed to polar extractions [71,72]. Similar results were reported by Zhou et al. [78] when evaluating the cytotoxicity of ocotillo derivatives using in vitro tests against cell lines. Previous information makes it possible consider this plant source for in vivo systems studies to demonstrate its effectiveness. In this sense, this shrub could be considered a potential antimicrobial source for treating respiratory tract infections.

### 3.7. Prosopis glandulosa

It is known as “honey mesquite” and is native to southwestern of Mexico and USA, growing mainly in the hyper-arid ecosystems [82]. This plant grows as a spiny shrub or small tree reaching 9 m height and shows bipinnately compound leaves [83]. In addition, its flowers presented a cylindric inflorescences of greenish-yellow color and fruit grow as linear indehiscent pods, straight or slightly curved with seeds and straw color in maturated conditions [83]. One of the main uses of *P. glandulosa* in local groups is by direct intake as food, using the seeds as floor [84]. Additionally, several ethnic groups use different parts *P. glandulosa* plant, such as leaves and bark, against different illnesses such as cough, sore throat, infections, inflammation, breast cancer, diabetic mellitus, muscular pain, eye wash, kidney stones, dyspepsia, eruptions, hernias, skin, and umbilical ailments [83,85,86]. On the other hand, scientific studies have demonstrated several biological activities of *P. glandulosa* extracts/compounds. Biological activities observed included anticancer, antihypertensive, antidiabetic, antimalarial, antihyperlipidemic, antileishmanial, cardioprotective, and immunostimulant; being the most recognize and outstanding, the antimicrobial effect [85,86,87,88,89]. 

Regarding to the antimicrobial effect of *P. glandulosa*, several studies have demonstrated its effectiveness against different microorganisms associated with respiratory tract infections. A methanol extract of *P. glandulosa* exhibited the capacity to inhibit the bacterial growth of *P. aeruginosa* (45%) and *S. aureus* (49%) according to the control. Additionally, it was also observed effect against opportunistic fungi cause of respiratory infection, such as *A. niger* (methanolic extract showed 88% of inhibition growth) and *Fusarium solani* (methanol and hexane extract showed 92% and 94%, respectively, of growth inhibition) [90]. In a study performed by Moorthy and Kumar [89], ethanol extract from *P. glandulosa* leaves showed growth inhibition of *S. aureus* (17.4 mm) and *Cryptococcus neoformans* (30.6 mm), compared to the control. Similarly, Imam et al. [91] observed that essential oil obtained from *P. glandulosa* seeds had the capacity to affect the growth of *S. aureus* (MIC: 3 µg/mL), *K. pneumoniae* (MIC: 5.5 µg/mL), *A. niger* (MIC: 34.5 µg/mL), and *F. oxysporum* (MIC: 20.5 µg/mL). López-Anchondo et al. [92] performed a leave extract from *P. glandulosa* which showed the capacity to affect the mycelial growth of different opportunistic fungi associated with a respiratory infection, such as *F. oxysporum* (5% inhibited 65% of mycelial growth), *Rhizopus oryzae* (5% inhibited 60% of mycelial growth), and *R. stolonifer* (5% inhibited 57% of mycelial growth). On the other hand, the antiviral effect of *P. glandulosa* has not been demonstrated; however, other species of the gender showed this effect [93]. The antimicrobial effect of *P. glandulosa* could be associated with different bioactive compounds identified in the extracts, such as flavonoids, phenolic acids, and alkaloids [94,95,96]. In this regard, Rahman et al. [85] isolated two alkaloids from ethanol extracts from *P. glandulosa* leaves with antimicrobial potential, which were identified as Δ1,6-juliprosopine and julirposine. Both compounds, showed antibacterial effect against *S. aureus* (MIC: 10 and 5 µg/mL, respectively), methicillin-resistant *S. aureus* (MIC: 10 and 5 µg/mL, respectively), and *M. intracellulare* (MIC: 10 and 2.5 µg/mL, respectively). An effect against *C. neoformans* was also observed (MIC: 1.25 and 0.63 µg/mL, respectively). In this way, it is important to highlight that both alkaloids showed equal or low MIC values than amphotericin B (MIC: 1.25 µg/mL), one of the most potent antifungals used in clinical practice. Additionally, the alkaloids did not exhibit a cytotoxic effect against VERO cells at the highest tested concentration (23,800 ng/mL). Ashfaq [97] used a root extract from *P. glandulosa* to isolate an alkaloid, prosopilosidine, which was evaluated against *C. neoformans* in vivo (murine model). They observed that the intraperitoneal administration (0.0625 mg/kg) reduced the fungal load by 76% compared with amphotericin B (85% at 1.5 mg/kg) after 5 days of the infection. Similarly, the oral administration exhibited a similar result because the tested alkaloid decreased the fungal organism by 82% compared to fluconazole (90% at 1.5 mg/kg) after 5 days of the infection. Additionally, it was observed that the evaluated alkaloid did not exhibit a toxic effect at 50 mg/kg against HepG2 cells. Also, it was observed that a dose of 20 mg/kg did not modify the normal plasma chemistry profile of tested mice. The above suggests that *P. glandulosa* has an interesting antimicrobial potential against microorganism associated with respiratory infection. Thus, the low cytotoxicity makes this plant an important alternative to develop antimicrobial agents against the mentioned pathogens.

## 4. Mechanisms of Action of Bioactive Compounds and/or Crude Extracts against the Disease

Plants contain chemical substances known as bioactive compounds, which can have a medicinal effect on the body due to their pharmacological activity. The main chemical groups of active drug components under broader heads are heterosides (e.g., anthraquinones, cardiac glycosides, cyanogenics, coumarins, phenols, flavonics, ranunculosides, saponosides, sulfurides), polyphenols (e.g., phenolic acids, cumarins, flavonoids, lignans, tannins, quinones), terpenoids (e.g., essential oils, iridoids, lactones, diterpones, saponins), and alkaloids (atropine, cocaine, daturin, hiosciamin, lysergic acid, nicotine, quinine) [98]. Mucilage and gums are heterogeneous polysaccharides, formed by different sugars, in general, they contain uronic acids. Other relevant active constituents in plants, such as vitamins, minerals, amino acids, carbohydrates and fibers, some sugars, organic acids, lipids, and antibiotics, are essential nutrients [44]. Flavonoids are a group of bioactive compounds that have antioxidant, antibacterial, antiviral, anti-inflammatory, antiangionic, analgesic, and antiallergic properties. On the other hand, they have also been found to have mutagenic activities and/or prooxidant effects. The human body contains numerous proteins with diverse functions. It has been studied that proteins Cytochromes P450 (CYPs) are a group of proteins that are distributed in many organs of the body and interact with flavonoids [45]. These compounds induce or modulate their metabolic activity. Flavonoids present in food are considered non-absorbable since they are attached to saccharides as beta-glucosides. However, the intestinal flora is responsible for hydrolyzing them into free flavonoids (aglycones), in this chemical form is easier to pass through the intestinal wall. However, quercetin, an onion glycoside absorbs even better than pure aglycone. The mechanism of action of flavonoids against microbial growth involves altering the plasma membrane, inducing mitochondrial dysfunction, and inhibiting key cellular process including cell wall formation, cell division, RNA, and protein synthesis, as well as efflux-mediated pumping system [99]. Furthermore, studies have shown that certain bioactive compounds, such as Narcissin, can suppress neutrophil infiltration and the activity of immune cells (CD3+/CD4+, CD3+/CD8+, and Gr-1+/CD11b) in bronchoalveolar lavage fluid (BALF) and lungs [100,101]. Ellagic acid has been found to stimulate enzyme responses like SOD and CAT, thereby attenuating pulmonary emphysema by inhibiting ROS, reducing lipid peroxidation, and enhancing antioxidant activity [14,102,103,104]. Carvacrol disrupts bacterial cell membranes, increases membrane permeability, and decreases cytoplasmic pH, thereby affecting the respiratory system [104,105,106,107]. Besides, additional compounds are listed in Table 2.

The plants discussed in this review contain a large amount of these compounds that have antioxidant function and intervene in various ailments including respiratory diseases. Table 2 shows several bioactive compounds that have been shown to have effects on respiratory system diseases, these compounds have been detected in plants from arid and semi-arid zones of Mexico. To date, many metabolic pathways that explain the action of the active compounds isolated from the plants of the Mexican semi-desert in different respiratory diseases are unknown. 

In Table 3 is presented both endemic plants and others with development in the arid and semi-arid zones of Mexico, and their effectiveness against respiratory diseases, doses, and some mechanisms of action. 

**Figure 3:**
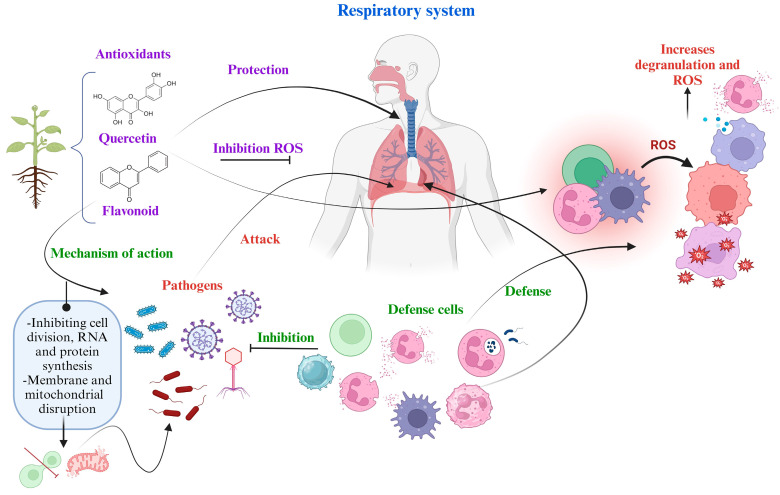
General mechanism of action of medicinal plants and cell defense against pathogens and ROS.

The human can avoid diseases due to the protection provided by the immune system. In a process of microbial infection or oxidative stress, defense cells are activated, also the synthesis of pro-inflammatory cells such as macrophages, interleukins, neutrophils from the MAPK metabolic pathway, which helps to inhibit pathogens. However, exposure of these cells to ROS leads to their lysis and degranulation, resulting in an increase in ROS level [131]. At this point, an important factor of protection is antioxidants from medicinal plants, which can inhibit ROS. In plants from arid and semi-arid zones of Mexico, the climatic factor can favor the expression of secondary metabolites, the effectiveness of their compounds and the description of some metabolic pathways on diseases of the respiratory system, which have been mentioned in Table 2 and Table 3. In the Figure 3, the general defense mechanisms available to humans against respiratory system diseases are presented. Bioactive compounds, such as antioxidants, exert their mechanism of action on bacteria, viruses, and fungi by inhibiting their development through the disruptions in cell division, mitochondria, protein synthesis, RNA, and ROS.

## 5. Ethnopharmacology of Plants from Arid and Semi-Arid Zones of Mexico

Traditional medicine continues to be a good alternative for healthcare in Mexico, particularly in rural regions where access to conventional medical services and medications is limited. Its utilization not only addressed gaps in healthcare accessibility but also contributes to reducing the reliance on antibiotics and other allopathic treatments [132]. An ethnopharmacological study carried out in Mexico revealed that 54% of healthcare professionals and 49% of physicians have integrated medicinal plants into their alternative practices [132].

Ethnopharmacology serves as an important link between cultures preserving ancestral knowledge of medicinal plants as healing practices and pharmacology as a science field that standardizes and regulates these remedies. The interdisciplinary approach facilitates the evaluation of the biological efficiency of traditional medicines and enables the development of new therapeutic interventions. Table 4 presents a comprehensive overview of native plants endemic to the arid and semi-arid regions of Mexico, outlining their traditional uses and effectiveness in treating respiratory ailments.

## 6. Commercially Available Important Plants in Mexico

In Mexico, there is a variety of commercially available herbal remedies, although only a limited number have received sanitary registration approval from the Federal Commission for Protection against Health Risks (COFEPRIS). As of 2023, one such approved herbal remedy is *Aloe vera*, commonly grown throughout the country and commercially marketed as Restaude [140]. Available in gel form, this pharmaceutical product is primarily designed to treat cosmetic skin concerns like acne scars and is also recognized for its efficacy in treating burns. In addition, beyond its known applications, *A. vera* has undergone research for additional therapeutic uses. Molecular docking studies have identified feralolide, also known as or ligand 6, as the most promising candidate among ten isolated compounds from *A. vera*, exhibiting significant reactivity with COVID-19 main protease (Mpro) [141].

Another notable example is the homeopathic medicine Simplex-Jet-Lag, approved by COFEPRIS in 2020) [140]. Formulated with *Passiflora*, *Valeriana Arnica montana*, this product is indicated for managing stress-related conditions and insomnia. While these plants are distributed in the South United States, Central America, and South America, their therapeutic effects extend beyond their common use. Despite its common use to treat anxiety and insomnia, *Passiflora* has a compound named Vitexin (apigenin-8-C-β-D-glucopyranoside), a flavone glycoside, which has demonstrated efficacy in reducing pulmonary edema and protein concentration in the alveoli [142]. Valerian extract has been found to modulate LL-37 gene and protein expression in lung cells, thus enhancing the immune system’s response to respiratory infections, including COVID-19 [143]. Additionally, *Arnica* exhibits bronchodilator activity like salbutamol, a well-known antiasthmatic medication [144].

## 7. Final Remarks

Respiratory diseases pose a significant global health challenge, affecting millions of individuals and causing substantial morbidity and mortality, particularly in underserved regions lacking adequate healthcare infrastructure. Among these, the emergence of the SARS-CoV-2 virus has underscored the urgent need for effective treatments, as it continues to claim countless lives worldwide. With no specific pharmaceutical intervention available for this novel virus, the exploration of alternative therapies has garnered increasing attention. 

Medicinal plants, deeply rooted in the traditions of ancestors and indigenous communities, offer promising opportunities for the management of respiratory diseases alongside conventional medicine. In Mexico’s arid and semi-arid zones, numerous plant species have been scientifically documented for their pharmacological efficacy against respiratory ailments. These plants not only demonstrate potent anti-inflammatory, antimicrobial, and anticancer properties but also harbor specific bioactive compounds with proven therapeutic benefits.

Further studies are needed to comprehensively understand various aspects: efficacy, optimal doses, bioactive compounds’ mechanisms against respiratory diseases, drug interactions, combining these plants with conventional medicine, potential side effects, action mechanisms against pathogens, and health regulations. While research suggests potential in treating respiratory diseases using plants from Mexico’s arid and semi-arid zones, more studies are warranted in the aforementioned areas. Their proven bioactive compounds’ antimicrobial and antiproliferative effects, coupled with traditional use, highlight them as a promising area for future medical research.

## Figures and Tables

**Table 1:** Factors involved in respiratory diseases.

Respiratory Diseases	Infection Factors	Climatic/Pollution and Other Factors	References
Bronchial asthma	Rhinoviruses, influenza virus, and respiratory syncytial virus	Cold air can exacerbate asthma symptoms by triggering the release of inflammatory mediators from mast cells, leading to airway contraction. Additionally, factors such as fatigue, infection, stress, and allergen exposure can further exacerbate asthma symptoms.	[31,32]
Chronic obstructive pulmonary disease COPD	A chronic lung disease characterized by airflow limitation. Some factors are respiratory pathogens (*Haemophilus influenzae*, *Streptococcus pneumoniae*, and *Moraxella catarrhalis*).	Environmental pollutions, cigarette smoke, anxiety, depression, and other factors contribute to an increase in airway inflammation.	[33,34,35]
Lung cancer	Pneumonia and tuberculosis. Idiopathic interstitial pneumonia has been linked to lung carcinogenesis, with causative agents including *Mycobacterium tuberculosis* or *S. pneumoniae*.	Tobacco smoking is the primary risk factor responsible for 80 to 90% of lung cancer diagnoses.	[36,37,38]
Pneumonia	Pneumococcal disease, commonly caused by *S. pneumoniae*, affects lungs (pneumonia), bloodstream (bacteremia), and the tissues and fluids surrounding the brain and spinal cord (meningitis).	Microorganisms are the main factor of respiratory diseases, with their prevalence influenced by seasonal climates. *S. pneumoniae* is more common in winter, while *Legionella pneumophila* predominates in summer.	[36,39,40]
Tuberculosis	Infectious disease transmitted through cough aerosols and is caused by *M. tuberculosis*. While it primarily affects the lungs, it can also elevate the risk of developing lung cancer.	There is a positive correlation between climate change and increased susceptibility to tuberculosis, particularly in developing countries.	[38,41]
Bronchitis	Viruses are responsible for about 90% of acute bronchitis cases, with multiple viruses implicated including RSV, human rhinovirus, parainfluenza virus, human metapneumovirus, coronavirus, adenovirus, influenza, and enterovirus.	Exposure to air pollutants can lead to decreased absorption rates in the airways, potentially compromising the individual’s immune system and increasing susceptibility to acute infections.	[42,43,44,45]

**Table 2:** Efficacy of bioactive compounds derived from plants in arid and semi-desert regions of Mexico against respiratory diseases.

Compound	Examples of Medicinal Plants	Effectiveness in Respiratory Disease	Description Compound	Chemical Structure	References
Quercetin	*Opuntia humifusa*; *O. dillenii*; *Larrea tridentata*; *Flourensia cernua*; *Turnera diffusa*; *Salvia officinalis*.	Potential against influenza A virus (IFV-A), rhinovirus, Respiratory Syncytial Virus (RSV). Inhibitory effect against pneumonia pathogens like *Moraxella catarrhalis*, *K. pneumoniae*, *S. pneumoniae*, and *P. aeruginosa*. Also exhibits anti-asthmatic efficacy.	It is one of the most abundant flavonoids. It exhibits antibacterial, antiviral, antioxidant, protein kinase inhibition, antineoplastic properties, and acts as a free radical scavenger.	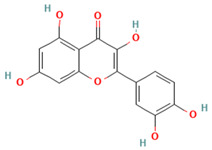	[21,66,102,108,109,110,111,112]
Rutin	*Schinus molle*; *Prosopis laevigata*; *P. glandulosa*; *O. dillenii*	Effective against avian influenza virus, herpes simplex virus 1 and 2 (HSV-1, HSV-2), and parainfluenza-3 virus. It inhibits essential proteins of SARS-CoV-2.	It is a flavonoid with potent antioxidant properties and is widely utilized in medicine. Besides, it shows antiprotozoal, antibacterial, and antiviral properties.	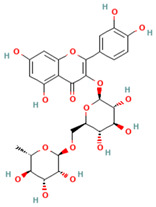	[21,85,110,113,114,115,116]
Ellagic Acid	*Fouquiera splendens*; *F. cernua*; *L. tridentata*	It exhibits anti-proliferative, anti-inflammatory, and antioxidant effects, stimulating the activity of SOD and CAT enzymes. It mitigates pulmonary emphysema by inhibiting ROS, reducing lipid peroxidation, and enhancing antioxidant defenses.	It is a trihydroxybenzoic acid, primarily known for its antioxidant and anti-proliferative effects in therapeutic action.	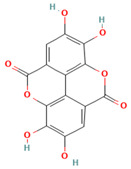	[14,102,103,104]
Juliprosopin	*P. glandulosa*; *P. flexuosa*	Effective against respiratory disease-causing microorganisms such as *S. aureus*, *M. intracellulare* and *C. neoformans*.	Alkaloid with antimicrobial potential	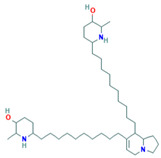	[85]
Nordihydroguaiaretic acid (NDGA)	*L. tridentata* and *L. divaricata*	Antibacterial effect against *S. aureus*, *S. pneumoniae*, and antiviral against *H. influenzae*. Lung cancer	It has a role as an antioxidant, anti-inflammatory, antitumoral, ferroptosis inhibitor, lipoxygenase inhibitor, and geroprotectant. It is found in various molecules including catechols and lignans.	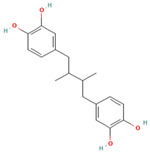	[46,104,117,118]
Thymol	*L. graveolens*; *Lavandula angustifolia*; *L. tridentata*	It has been utilized for its antiseptic, antibacterial, and antifungal actions in respiratory system diseases. In *P. aeruginosa* and *S. aureus*, it disrupts the cell membrane, increases membrane permeability, and decreases cytoplasmic pH in these bacteria.	It is a phenolic compound, a monoterpene derived from cymene. It acts as a volatile component present in the oils of various vegetal plants.	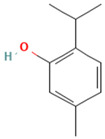	[104,105,106,107]
Carvacrol	*L. graveolens*; *L. angustifolia*; *L. tridentata*	In *P. aeruginosa* and *S. aureus*, it disrupts the cell membrane, enhances membrane permeability, and reduces cytoplasmic pH in these bacteria.	It is a type of phenol with antimicrobial and anti-inflammatory properties, capable of inhibiting the production of microbial toxins.	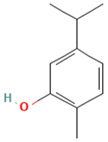	[104,105,106,107]
3′-Demethoxy-6-O-demethylisoguaiacin	*L. tridentata*	Effective against methicillin-resistant *S. aureus* (MRSA) and exhibits moderate activity against a drug-resistant strain of *M. tuberculosis*.	Antibacterial, antiprotozoal, anthelmintic, antifungal, antiviral, anticancer, and antioxidant activities. It disrupts bacterial ABC transporters, affecting substrate transport across the membrane.	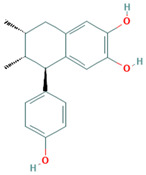	[119,120]
β-sitosterol	*J. dioica*	Effective against *Cryptococcus neoformans*; the symptoms produced by this microorganism are pneumonia and meningitis.	A high antibacterial and antifungal activity.	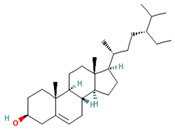	[55]
Eucalyptol or 1,8-Cineole	*T. diffusa*, *Poliomintha longiflora*; *L. graveolens*; *S. officinalis*.	This compound has antitussive effects, regulates mucus hypersecretion and asthma by inhibiting anti-inflammatory cytokinins, and reduces inflammation and pain when applied topically.	It is a monoterpene with antibacterial and antioxidant properties. Eucalyptol has the ability to penetrate the blood–brain barrier.	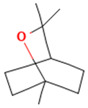	[57,112,121,122]
p-cumaric acid/Coumaric acid	*Fouquieria* spp.; *F. cernua*; *S. officinalis*.	Attenuates lipopolysaccharide-induced pulmonary inflammation in rats (from Gram-negative bacteria). It also exhibits antibacterial effects against *M. tuberculosis*.	It is a flavonoid and one of the three isomers of hydroxycinnamic acid. It has biochemical activities such antioxidant, an antimutagenic, and anti-ulcer properties.	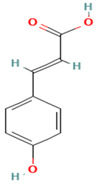	[111,123]
Caffeic acid	*O. dillenii*; *F. cernua*; *S. officinalis*.	Regulates the proliferation, migration, and apoptosis of lung cancer cells.	It is a hydroxycinnamic acid derivative, namely cinnamic acid, with potential antioxidant, anti-inflammatory, and antineoplastic activities.	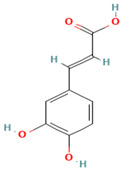	[102,110,124]
Linalool	*L. angustifolia*	Bactericidal activity against *K. pneumoniae*	It is a monoterpenoid, a volatile oil component, and an antimicrobial agent.	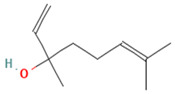	[106]
β-Caryophyllene	*F. cernua*; *P. longiflora*; *L. graveolens*; *S. officinalis*.	Effective against *Mycoplasma pneumoniae*.	It is a sesquiterpene with anti-inflammatory properties.	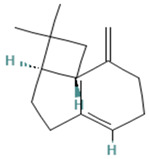	[102,112,122,125,126,127]

**Table 3:** Endemic and locally adapted plants from arid and semi-desert regions of Mexico, their effectiveness against respiratory diseases, doses, and mechanisms of action.

Plant	Part Used	Extraction Solvent	Dose	Active Compound Isolated	Mechanism of Action	Activity	References
*Larrea tridentata*	Leaves	Chloroformic (CLO) and methanol (MET) extracts	MIC 80 = 31.25 μg/mL against *S. pneumoniae*	Antioxidant NDGA	Unspecified	The MET and CLO extracts of *L. tridentata* were also effective against *S. aureus*, *S. pneumoniae*, and the MET was also active against *H. influenzae*.	[46,117]
*Petiveria alliacea*	Leaves	Metanol extract	Oral gavage to mice at 100–400 mg/kg body weight once daily from days 18 to 23.	No active compound was reported, but the extract exhibited high antioxidant activity against DPPH radical scavenging.	Inhibited the production of chemokines, eotaxin, TNF-α, IgE, TGF-α, IgE, and TGF-β1. In addition, it reduced cytokine levels of IL-4, IL-5, IL-13, and ICAM-1 in bronchoalveolar lavage fluid.	Administration could inhibit airway inflammation, regulate cytokines and chemokines, and improve pulmonary conditions in an allergic murine model of asthma.	[128]
*Fouquieria splendens*	Leaves	Methanol extract	MIC 25.0 μg/mL in *S. aureus*	ND	Unspecified	Antimicrobial activity against *S. aureus* and *K. pneumoniae*	[75]
*Leucophyllum frutescens*	Leaves	Methanol extract	MIC 25.4 μg/mL in *S. aureus*.	ND	Unspecified	Antimicrobial activity against *M. tuberculosis, S. aureus*, and *H. influenzae* b type	[75]
*L. frutescens*	Roots and leaves	Methanol extract	MIC 62.5 (roots), 125 (leaves) g/mL	ND	Unspecified	Antimicrobial activity against the drug-resistant strains of *M. tuberculosis*	[129]
*Chrysanctinia mexicana*	Roots	Ethyl ether extract	MIC 62.5 g/mL	ND	Unspecified	Antimicrobial activity against the drug-resistant strains of *M. tuberculosis*	[129]
*Cordia boissieri*	leaves	Methanol extract	MIC250 g/mL	ND	Unspecified	Antimicrobial activity against the drug-resistant strains of *S. aureus*	[129]
*Schinus molle*	fruits	Hexane-based extract	MIC 62.5 g/mL	ND	Unspecified	Antimicrobial activity against the drug-resistant strain of *Streptococcus pneumoniae*	[129]
*Opuntia ficus indica (OFI)*	Stems	OFI extracts (water, 30% ethanol, or 50% ethanol extracts)	100 and 200 mg/kg OFI extracts	Narcissin in 50% ethanol extracts	All extracts suppressed neutrophil infiltration and the number of immune cells (CD3+/CD4+, CD3+/CD8+, and Gr-1+/CD11b) in bronchoalveolar lavage fluid (BALF) and lungs. OFI extracts also decreased the expression of cytokines and chemokines, including chemokine, including interleukins, macrophage inflammatory protein-2, and tumor necrosis factor (TNF)-α.	OFI extracts may be used to prevent and treat airway inflammation and respiratory diseases.	[100]
*Eysenhardtia texana*	Leaves	Methanol-dichloromethane extract	0.1 mg/mL	4′,5,7-trihydroxy-8-methyl-6-(3-methyl-[2-butenyl])-(2S)-flavanone, 4′,5,7-trihydroxy-6-methyl-8-(3-methyl-[2-butenyl])-(2S)-flavanone	Flavonoids often inhibit fungal growth through diverse mechanisms, such as disrupting the plasma membrane, inducing mitochondrial dysfunction, and inhibiting processes like cell wall formation, cell division, RNA and protein synthesis, and the efflux-mediated pumping system.	Activity against *S. aureus* and inhibited the growth of *Candida albicans*	[99,130]
*Carya illinoensis*, *Jatropha dioica*, *Selaginella lepidophylla*, *Euphorbia antisyphilitica*	Leaves	Methanol extracts	MIC 500 µg/mL and LD50 of 1000 µg/mL.	Phytochemical tests were positive for flavonoids, lactones, quinones, triterpenes, and sterols.	Unspecified	Activity against *S. aureus* and *K. pneumonia*	[49,52]
*Turnera diffusa*	Leaves	Ethanol/distilled water/glycerol (63/27/10)	MIC 300 µg/mL	Flavonoids, phenolic acids and derivatives, cyanogenic glycosides, fatty acids, alkaloids, and sugars conjugates.	Unspecified	Activity against *S. aureus*	[57,59]
*Fouquieria splendens*	Leaves	Methanol extract	(C33-A IC50: 9.06 µg/mL; HeLa IC50: 74.7 µg/mL	Phenolic compounds, ellagic acid, kaempferol-3-β-glucoside.	Unspecified	Anti-proliferative effect specifically against cervical cancer cell lines, particularly HPV negative cells	[72]

N.D. = Not detected.

**Table 4:** Ethnopharmacology of plants from arid and semiarid zones in Mexico.

Scientific Name	Family	Common Name	Parts Used	Form of Use	Ailment or Symptoms	References
*Bougainvillea glabra*	Nyctaginaceae	Bugambilia	Leaves/Flower	Infusion/oral	Asthma/Cough/Bronchitis	[132,133]
*B. spectabilis*	Nyctaginaceae	Bugambilia	Leaves/Flower	Infusion/oral	Snoring or lung pain/Flu/Bronchitis	[133]
*Lippia berlandieri*	Verbenaceae	Oregano	Whole plant	Infusion/oral	Cough	[132]
*Ruta chalepensis*	Rutaceae	Ruda	Whole plant	Infusion/oral	Cough	[132]
*Mentha spicata*	Lamiaceae	Hierbabuena/menta	Whole plant	Infusion/oral	Bronchitis/Cough	[129,132]
*Foeniculum vulgare*	Apiaceae	Hinojo	Whole plant	Infusion/oral	Bronchitis	[132]
*Schinus molle*	Anacardiaceae	Pirul	Leaves	Infusion/topical	Cough	[132]
*Allium cepa*	Alliaceae	Cebolla	Bulb	Bulb/bulb infusion	Tuberculosis/Cough	[132]
*Prosopis juliflora*	Fabaceae	Mesquite	Bark/leaves	Paste/poultice/Gum/Smoke/Decoction/Infusion/Maceration/Baths	Respiratory disorders/Asthma/Antibacterial capacity against Gram-negative bacteria	[134,135]
*Prosopis* spp.	Fabaceae	Mesquite	Bark/Resin from the trunk/Seed pods	Resin from the trunk/Seed pods/leaves.	Spasmolytic/Bronchodilator/Vasodilator/Asthma/Sore throat/Antibacterial	[134,135,136]
*Larrea tridentata*	Zygophyllaceae	Gobernadora, hediondilla or guamis, creosote bush	Leaves	Infusion/Oral	Cold virus infections e.g influenza virus /Sinusitis/Lung cancer	[117,118]
*Jatropha dioica*	Euphorbiaceae	Sangre de drago or Sangregrado	Root/Stem	Infusion/Decoctions/Oral	Antimicrobial/asthma/Influenza type A virus	[118]
*Turnera diffusa*	Turneraceae	Damiana, hierba del venado, hierba de la pastora	Leaves	Infusion/Decoctions/Oral	Bronchitis/Cough/Pulmonar/Respiratory diseases/Expectorant	[47,118]
*Opuntia ficus-indica*	Cactaceae	Nopal, penca, tunera.	Dry flowers	Infusion/Decoctions/Oral	Antiviral, RSV. Inhibitory effect against pneumonia pathogens.	[137]
*Fouquieria splendens*	Fouquieriaceae	Ocotillo	Fresh flowers/Seeds	Infusion/Oral	Cough/Sore throat	[138]
*Chaenopodium ambrosioides*	Chenopodiaceae	Epazote	Flowers/Leaves/Stems	Infusion/Oral	Flu/cold/asthma and pectoral complaints	[129]
*Flourensia cernua*	Asteraceae	Hojasen	Leaves/Stems	Infusion/Oral	Expectorant/Bactericidal compounds against multidrug-resistant *M. tuberculosis*	[129]
*Lavandula angustifolia*	Lamiaceae	Lavanda	Flowers/Leaves/Stems	Infusion/Oral	Infection by *K. pneumoniae*	[106]
*Gnaphalium oxyphyllum*	Asteraceae	Gordolobo	Flowers/Leaves/Stems	Infusion/Oral	Cough/Chest pain/Bronchitis/Sore throat/Gripe/Asthma/Cancer	[139]

## Data Availability

Not applicable.
